# Empowering digital public health: a study on the mechanisms by which the application of digital technology drives social entrepreneurial intentions in the health sector among university students

**DOI:** 10.3389/fpubh.2026.1856541

**Published:** 2026-07-17

**Authors:** Zhen Wang, Zhouyan Zheng, Lulu Song

**Affiliations:** Zhejiang University of Finance & Economics Dongfang College, Haining, China

**Keywords:** digital health practice literacy, digital public health, digital technology, psychological capital, social entrepreneurial intention

## Abstract

Amidst the tide of the rapid development of digital public health, digital technology has become a core tool driving university students to address social pain points such as health inequality and engage in social innovation. Based on social innovation theory, this study constructs a multiple mediation model of “digital technology application––digital health practice literacy––psychological capital––social entrepreneurial intention,” aiming to explore the internal capability and psychological transmission pathways through which digital technology empowerment influences university students’ social entrepreneurial intention. Using questionnaire data from 276 students and structural equation modeling with Bootstrap testing, results indicate: (1) digital technology directly enhances social entrepreneurial intention; (2) both digital health practice literacy and psychological capital independently mediate this relationship; and (3) a sequential pathway exists whereby digital health practice literacy strengthens psychological capital, further reinforcing entrepreneurial intention. This study clarifies the dual “capability–mindset” mechanism of digital empowerment, extends research on digital health-driven social innovation, and provides actionable insights for universities to cultivate digitally proficient, psychologically resilient social entrepreneurs.

## 1 Introduction

Against the broader backdrop of the ongoing deepening of the global digital health transformation, society is also entering a VUCA (Volatility, Uncertainty, Complexity and Ambiguity) era marked by the intertwining of complexity and uncertainty (1). From structural imbalances in public health systems to social divides arising amid the dividends of digital health, traditional governance models can no longer independently support the demands of complex health interventions. Existing research has extensively discussed digital health development and social entrepreneurship, yet a systematic literature review on the integrated mechanism of digital technology application, digital health practice literacy, psychological capital, and social entrepreneurial intention remains insufficient, which constitutes the research gap addressed by this study. In this context, social innovation, as a paradigm that meets urgent public health needs by reconstructing social relations and optimizing resource allocation, is gradually moving from the margins to the core of public policy and health development (2). Social innovation theory suggests that the driving force of innovation is shifting downward from macro-level organizations to highly agentic individuals (3). As the group with the greatest innovative potential and the highest sensitivity to health issues in society, college students’ social entrepreneurial intention is not only a buffer for easing employment pressure, but also a key engine for creating public health value (4). According to theories related to entrepreneurial psychology, university students’ social entrepreneurial intention is a comprehensive psychological tendency at the cognitive, emotional, and volitional levels, and also a core antecedent variable for predicting entrepreneurial behavior (5). However, health-related social entrepreneurship in the traditional sense faces significant dual barriers in both resources and psychology. On the one hand, identifying hidden social pain points requires extremely strong information integration capabilities; on the other hand, the complexity of the entrepreneurial pathway poses severe challenges to individuals’ resilience and adaptability.

As digital technology applications, serving as a general-purpose technology, comprehensively penetrate various social fields such as public health, education, and entrepreneurship, they are reshaping individual innovative behavior patterns and the logic of public health value creation (6). Public health entrepreneurship provides a new path for translating health knowledge into sustainable intervention programs, and the popularization of digital technology is fundamentally shaking up the threshold of this social innovation and entrepreneurship. Digital technology applications, as a universal enabling foundation, affect more than just productivity; they profoundly reconstruct the cognitive microecology of college students. For college students, digital technology applications are not merely a simple tool substitution, but rather an all-around cognitive empowerment (7). By providing real-time data analysis, automated task processing, and predictive health decision support, digital technology applications essentially offer college students a “digital shock absorber.” This reliance on technology greatly alleviates the feelings of intimidation college students face when confronting complex health-related social entrepreneurship, thereby enhancing their entrepreneurial confidence (8). Simultaneously, by reshaping an individual’s health cognition and sense of technological efficacy, digital tools become an important technological variable influencing entrepreneurial psychology and behavioral decision-making (9). Therefore, understanding how digital technology applications translate into college students’ social entrepreneurial intentions and motivations has become an urgent interdisciplinary proposition to be addressed in the fields of public health innovation and entrepreneurship education.

A review of the literature reveals that while academic discussions on digital technology-driven entrepreneurship have experienced explosive growth, in-depth empirical research on the ecosystem of public health social entrepreneurship among university students remains fragmented and uneven, failing to form a closed-loop theoretical explanatory framework. Existing studies on the mechanisms of digital technology empowerment often remain at the superficial level of a black box, lacking a refined depiction of the internal pathways of capability transformation. Some cutting-edge literature has confirmed a significant correlation between the application of digital technologies and university students’ social entrepreneurial intentions, and has attempted to introduce social innovation theory for preliminary explanation (10). However, these studies often treat the application of digital technologies as a homogeneous resource input (11), overlooking how it functions as an empowerment mechanism—reshaping individuals’ underlying mindset to trigger action intentions. Secondly, most current literature, when discussing digital capabilities, remains confined to traditional dimensions of information processing, failing to capture the crucial element of digital health practice literacy in the era of digital health, nor incorporating digital health applications into the core framework of capabilities (12, 13). Existing models exhibit a significant disconnect in studying the interaction effects between “technological tools” and “psychological energy.” Psychological capital, as an intrinsic motivational driver of entrepreneurial behavior, encompasses four core elements: self-efficacy, hope, optimism, and resilience. It has yielded substantial results in traditional entrepreneurship research (14) and has been confirmed as a key positive psychological predictor of university students’ entrepreneurial intentions (15). However, its patterns of change in the context of digital health interventions have yet to be fully explored. Although the study by Balgiu et al. (16) suggests that technology application may enhance entrepreneurial confidence by boosting self-efficacy, it fails to reveal the underlying logic of resilience in the face of health crises. Finally, existing research demonstrates a significant vacuum in the contextual application of “public health value creation.” Most studies on digital technology and entrepreneurship are situated in high-profit, high-competition commercial domains (17), resulting in minimal attention to public health social entrepreneurship. From the perspective of social innovation, university students’ entrepreneurial motivations often stem from interventions addressing health inequalities and social pain points (18). Yet, existing mediation models fail to explain how digital technologies empower university students to identify health-related social pain points and lack systematic examination of the “capability–psychology–intention” chain transmission mechanism (19).

To address these gaps, this study constructs a multiple mediation model of digital technology application––digital health practice literacy––psychological capital––social entrepreneurial intention, aiming to systematically reconstruct the driving mechanism of college students’ social entrepreneurial intention. This study seeks to answer how digital technology application drives the implementation of social innovation in the health field by reshaping the paradigm of college students’ public health practice abilities and enhancing their internal resilience. This not only provides a theoretical basis for the transformation of digital public health education in higher education institutions, but also offers a novel empirical footnote for understanding how digital technology acts as an endogenous driving force for social change in public health, possessing significant practical guiding implications.

## 2 Research hypotheses and model

### 2.1 Digital technology application and social entrepreneurial intention

Digital technology serves as a transformative force in shaping individual opportunity recognition and entrepreneurial motivation. The present study conceptualizes digital technology application as encompassing four principal categories: generative artificial intelligence (e.g., ChatGPT, Claude), data analytics tools (e.g., Python, SPSS, Google Analytics), digital collaboration platforms, and low-code productivity tools. These technologies collectively lower barriers to information processing, resource mobilization, and collaborative problem-solving in the public health domain. Social innovation theory provides a foundational lens for understanding this process. According to Mulgan (20), social innovation refers to innovative activities and services aimed at meeting social needs, usually disseminated by organizations with a primary social purpose, and it emphasizes a process that moves from “observation” to “prototyping” and then to “scaling up.” Social innovation theory also places strong emphasis on social capital as a driving force: social capital can directly and positively influence social innovation, and the efficiency of its cross-boundary circulation often determines whether social innovation succeeds (21, 22). From the perspective of capability equalization in social innovation theory, when innovation tools become inexpensive and readily accessible, more groups can become the main actors of innovation (23). As one of the most revolutionary general-purpose technologies of our time, digital technology breaks through the information barriers and professional thresholds embedded in traditional social structures, while also reconstructing the architecture of social innovation through technological democratization (24). Within the paradigm of social innovation theory, emerging digital technologies like artificial intelligence can be regarded as “digital weapons” that trigger social innovation and individual empowerment, possessing the capability to process vast and fragmented social data (25). When university students engage deeply with digital tools, their ability to identify social pain points is significantly enhanced, and they are endowed with a form of “digital capital” that enables them to move beyond class and capital constraints (67). As a result, when confronting challenges such as unemployment, mental health, and green development, they are less likely to feel helpless. Mair and Marti (26) argue that social entrepreneurial intention derives from a positive evaluation of the feasibility of social change. In this respect, the accessibility of digital technology reduces the entry barrier to entrepreneurship, enabling university students to be confident in their ability to turn complex social problems into business or social solutions and thereby creating a higher social entrepreneurial intention. Based on this logic, the present study formulates the following hypothesis:

H1: Digital technology application positively influences university students’ social entrepreneurial intention.

### 2.2 The mediating role of digital health practice literacy

The empowerment logic of social innovation theory accentuates that people’s power to effect change comes from their mastery over the tools of production (20). In the AI era, the application of digital technologies such as artificial intelligence has gone beyond the traditional “command-response” model of software and has transformed into a generative learning environment characterized by interactivity and inspiration (27). First, the frequent use of AI technology has prompted university students to achieve cognitive iteration in information filtering and evaluation. According to the empirical study by Rodrigues et al. (28), university students with deep exposure to digital technologies such as generative AI demonstrate stronger critical thinking when processing complex social information, thereby promoting the evolution of digital health practice literacy from basic information retrieval to advanced “human-machine collaborative creation” (69). Second, AI applications have lowered the threshold for acquiring complex skills, leading to a structural shift in the connotation of digital health practice literacy: students without technical backgrounds can also engage in preliminary coding or prototype design. Such frequent interactive practice not only improves students’ proficiency in operating digital tools, but also subtly cultivates their habit of using algorithmic logic to deconstruct social problems, thereby enabling leapfrog growth in digital health practice literacy. Social innovation theory holds that the essence of innovation lies in transforming new ideas into actions that solve social problems (20). As a core capability for individuals to create value in the context of digital-intelligent living, digital health practice literacy is the critical link connecting “technological cognition” and “entrepreneurial practice.” A high level of digital health practice literacy provides individuals with strong control over the allocation of digital resources. Individuals with high digital health practice literacy can more keenly use AI tools to identify vulnerabilities in social systems and can rapidly mobilize cloud-based resources to construct low-cost, high-efficiency digital governance solutions, greatly stimulating individual entrepreneurship (29). Moreover, the study by Battisti et al. (30) confirms that higher levels of digital literacy are associated with lower perceived difficulty in launching and conducting social entrepreneurship projects, meaning individuals feel more capable and less challenged when engaging in social entrepreneurial behavior. Therefore, digital health practice literacy is not only a competitive tool for university students in digitalized living, but also the logical mediating point through which they transform the dividends of AI technology into entrepreneurial decision-making. In summary, by constructing a generative environment for learning and experimentation, digital technology application accelerates knowledge acquisition and solution simulation, thereby enabling the systematic development of university students’ digital health practice literacy. This advanced digital health practice literacy, in turn, provides individuals with dual support in identifying social opportunities and entrepreneurial capability, ultimately driving the growth of social entrepreneurial intention. Accordingly, this study proposes the following hypothesis:

H2: Digital health practice literacy mediates the positive effect of digital technology application on university students’ social entrepreneurial intention.

### 2.3 The mediating role of psychological capital

Since innovation and entrepreneurship usually require undermining existing interest structures, we are subject to a great deal of social push back and psychological pressure. For this reason social innovation theory contends that successful innovators must be socially resilient (31). Psychological capital is a positive psychological state conditional on (independent of) self-efficacy, hope, optimism, and resilience. Self-efficacy, the core of psychological capital, is “an individual’s conviction that he or she can successfully execute the behavior required to produce the outcomes” (32). In the AI era, generations of college kids will work with generative AI through prompt engineering to quickly create product prototypes, draft business plans, or fuel analysis of complex policy text (33). The immediacy of feedback and swift experience of success made possible by digital technology greatly amplify students’ feelings of technological mastery. When students believe AI can “replace” their lack of professional experience, their self-efficacy in the context of social entrepreneurial tasks can increase significantly (34). Furthermore, the predictive analytic features of digital technology can analyze huge amounts of data and present students with numerous alternative solutions (35), allowing them to hold onto success expectations even in the face of social paradoxes and thus reinforcing the hope dimension of psychological capital as well as yielding more positive expectations concerning entrepreneurial career prospects. When individuals perceive the external technological environment as supportive, they in turn display elevated levels of dispositional optimism, and are more likely to consider entrepreneurship as a feasible career (36). Moreover, digital technology can also serve for virtual experimentation, market simulation and risk rehearsal, allowing university students to model the entrepreneurial process in a zero-loss setting and learn recovery mechanisms. Such simulated experience strengthens psychological resilience and enhances recovery capacity when confronting social resistance in real-life (37). In view of the above, this study argues that digital technology application is not only a productivity tool, but also a psychological scaffold, modifying to a substantial degree these university students’ stock of psychological resources, enhancing psychological capital, reducing fear and avoidance in the entrepreneurial process and promoting social entrepreneurial intention. This leads us to the following hypothesis:

H3: Psychological capital mediates the positive effect of digital technology application on university students’ social entrepreneurial intention.

### 2.4 The multiple mediating effects of digital health practice literacy and psychological capital

Social innovation theory suggests that innovation signifies not merely the restructuring of the objective material world, but also the “ecological evolution of the innovator’s own capabilities” (38). With a digital technology boom taking place, GI–students as an industrial force in social innovation and entrepreneurship are likely to develop their social entrepreneurial intention through a logical chain of “technology perception–capability acquisition–mindset strengthening–behavioral intention.” Social entrepreneurship rightfully calls for university students to possess strong information-processing capabilities and solution-building capability, and the diffusion of digital technology provides them democratized and low-cost technological support to achieve it. Through high-frequency technological interaction, students may elevate the textual content of digital health practice literacy from basic information searching to composite algorithmic collaborations and prompt-engineering competency (39). This improvement in turn signals a fundamental transition from passive technology users to active partners who learn and innovate alongside intelligent systems. By social innovation theory, growth in capability comes before psychological empowerment (40). When university students gain high levels of digital health practice literacy and ability to use digital technology for solving real problems, they attain sufficient feelings of technological efficacy to offset the frustration of entrepreneurship with feelings of technological control, thereby heightening psychological capital by the ability transfer affecting. Entrepreneurial intention is the taking on of responsibility for social problems (20). University students with high psychological capital are more socially committed; They are not satisfied with just achieving commercial success, but are more likely to use AI technology for social balancing. A high level of digital health practice literacy affords individuals the capacity for low-cost trial and error, while high psychological capital endows them with strong stress tolerance. This form of psychological empowerment directly pushes individuals from spectators to participants and forms a strong trigger of social entrepreneurial intention to the sense of mission for social innovation (41). Thus, the expression of digital technology application not only changes the external of the production environment but also triggers an internal evolutionary chain that constructs university students as the redemption of the “digitally intelligent innovators” character. We propose the following hypothesis:

H4: Digital health practice literacy and psychological capital serially mediate the positive effect of digital technology application on university students’ social entrepreneurial intention.

### 2.5 Research hypothesis model

**Figure 1:**
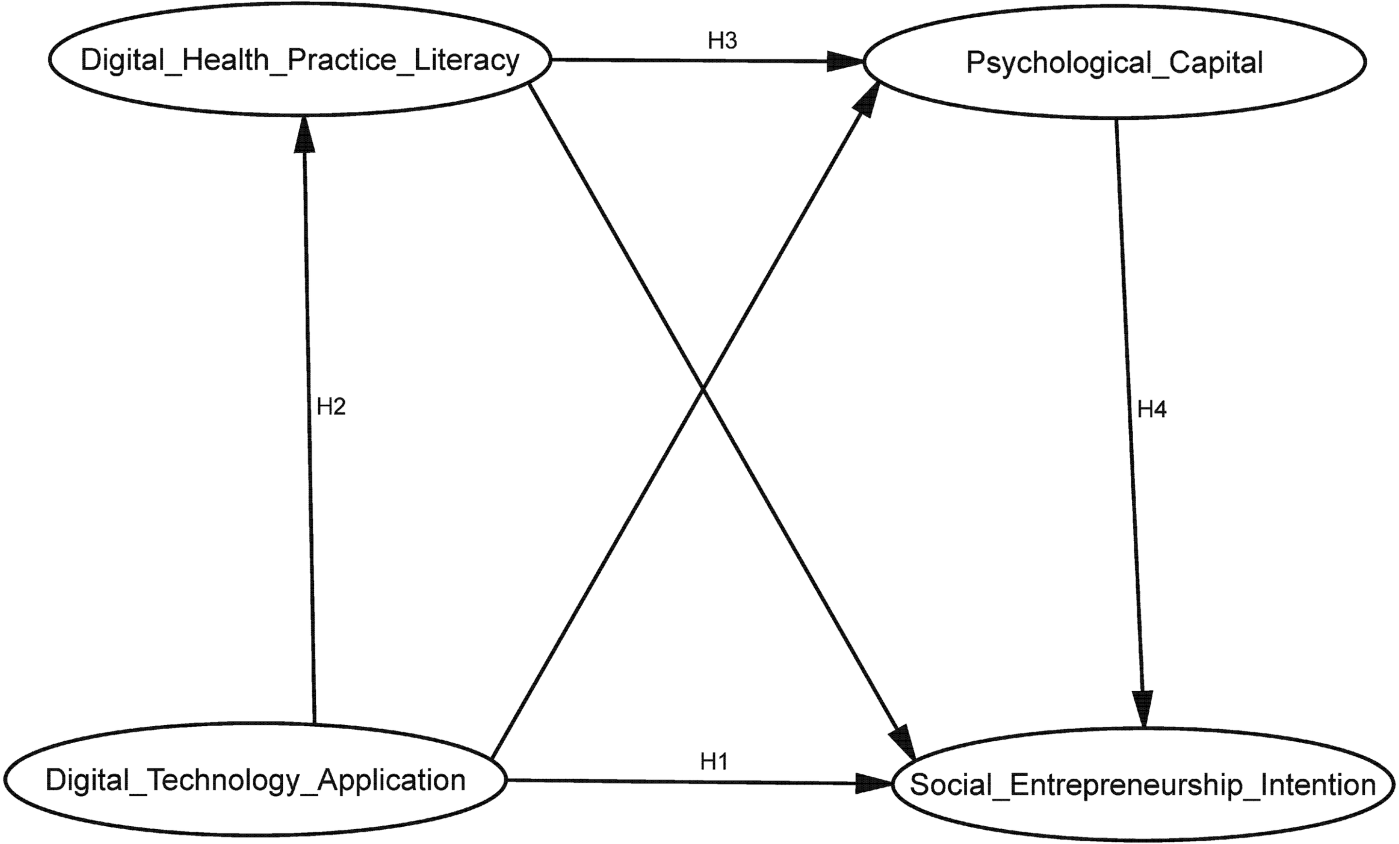
Research hypothesis model.

Based on the research hypotheses proposed above, this paper, grounded in social innovation theory, takes digital technology application as the independent variable, introduces digital health practice literacy and psychological capital as mediating variables, and uses social entrepreneurial intention as the dependent variable to construct a theoretical model of the impact of digital technology application on university students’ social entrepreneurial intention in the health field, as shown in Figure 1. To test this model, this study employs a structural equation model for data analysis and uses SPSS 31.0 and AMOS 28.0 software to fit and test the model. By testing this theoretical model, this study aims to go beyond a simple verification of direct effects and to explore in depth the mechanism through which digital technology application drives the formation of university students’ social entrepreneurial intention, thereby providing theoretical guidance and practical insights for entrepreneurship education in universities and innovation-driven social development.

## 3 Research method

### 3.1 Variable measurement

To improve the quality of the data collected, all scales adopted from previous valid instruments with high levels of reliability and validity. A seven-point Likert scale was adopted, where “1” indicates complete disagreement and “7” indicates complete agreement.

#### 3.1.1 Social entrepreneurial intention

This scale was adapted from the entrepreneurial intention questionnaire developed by Liñán and Chen (42). In light of the characteristics of university students, five items were included, such as “I am prepared in all respects to start a business,” “My future career goal is to become an entrepreneur,” “I will make every effort in the future to found and run my own company,” “I am convinced that one day I will establish my own enterprise,” and “I have seriously considered starting a business.”

#### 3.1.2 Digital technology application

This scale was developed with reference to the Unified Theory of Acceptance and Use of Technology (UTAUT) (43) to assess the driving influence of digital technology application. The present study conceptualizes digital technology application as encompassing four core categories: generative artificial intelligence (e.g., ChatGPT, Claude), data analytics tools (e.g., Python, SPSS, Google Analytics), digital collaboration platforms, and low-code productivity tools. Four items measured participants’ usage frequency and proficiency across these domains for entrepreneurship-related tasks, such as “I often use AI tools to assist in writing business plans or conducting market research” and “I can skillfully use digital technology to handle entrepreneurship-related daily tasks (e.g., data analysis, coding, and poster generation).”

#### 3.1.3 Psychological capital

This scale was based on the four-dimensional framework of positive psychological resources developed by Luthans et al. (44) and the Psychological Capital Questionnaire (PCQ), combined with the scale used by Yuan et al. (45) in their study of psychological capital and university students’ entrepreneurial intention. A total of 25 items were included, such as “When facing adversity in entrepreneurship, I actively seek support and summarize experience” and “I believe that I can make correct decisions regarding the setting of entrepreneurial goals.”

#### 3.1.4 Digital health practice literacy

This scale was adapted from Bachmann et al. (46) on digital competence and entrepreneurial intention and Ivanović et al. (47) on university students’ data-driven entrepreneurial competence. While these original instruments assess generic digital and business-analytic skills, the present study systematically reoriented all 21 items toward digital public health social entrepreneurship through expert panel review and pilot testing. To align with the digital public health context, items were shifted from generic market research and business planning toward competencies in identifying community health needs, analyzing health disparities, and designing digital health interventions. This ensures the scale captures the application of digital tools for public health problem-solving rather than broad commercial digital literacy. Moreover, to suit university students, items were calibrated to reflect accessible, entry-level practices using widely available digital platforms—such as social media, mobile health apps, and open datasets—rather than specialized clinical or advanced technical tools. This adjustment accommodates students’ high digital familiarity while remaining relevant to health-related social entrepreneurship. The adapted items were reviewed by public health and digital health experts for content validity and pilot-tested with 30 university students to ensure clarity and appropriate response distribution before the main survey.

Finally, the four research variables were compiled into a questionnaire entitled “A Survey on the Influence of Digital Technology Applications on University Students’ Social Entrepreneurship Intentions in the Health Sector.” The first half of the questionnaire through collection of basic information collected data of five control variables—gender, grade level, major, place of origin, and annual family income—as well as two other variables, participation in entrepreneurship course and entrepreneurial experience. The latter half contained the questions tapping digital technology application, social entrepreneurial intention, digital health practice literacy, and psychological capital.

### 3.2 Sample data statistics

Data were collected using a questionnaire survey; respondents were mainly students from universities in Hangzhou, Ningbo, and Jiaxing, Zhejiang Province. The online survey used a professional questionnaire platform, and respondents were screened by maximum-difference sampling; offline, purposive sampling was used, and questionnaires were directly distributed at some universities. A total of 359 questionnaires were collected; 15 duplicates by student ID were removed, as well as 68 questionnaires that had the same response for most items. Eventually 276 valid questionnaires were collected, and the effective recovery rate met the requirements of social science research. Descriptive statistical information is presented in Table 1. The numbers of male and female students accounted for 47.8 and 52.2% respectively, indicating a roughly even distribution of gender so that the findings cannot be prejudiced by any one gender or another. In regard to grade level, the sample included students from the full undergraduate cycle right up to postgraduate study, third-year undergraduates being in the largest numbers (72 students). The sample also covered multiple disciplinary categories. The ratio of the respondents’ origin of home was also well balanced: 37.0% from cities, 34.0% from county towns and townships, 29.0% from rural areas. So that the study can reflect the difference of entrepreneurial cognition and resource endowment under different growth environments. In addition, the number of valid respondents who attended entrepreneurship-related courses was 58.7%, the number of valid respondents who had actual entrepreneurial experience was 32.2%. Overall, most respondents had a basic level of entrepreneurial knowledge and practice, thus serving as a reasonable basis and good representative to study the mechanism through which digital technology application impacts social entrepreneurial intention.

**Table 1:** Descriptive statistics of the survey sample.

Basic category	Option	Frequency	Percentage (%)
Gender	Male	132	47.8
	Female	144	52.2
Grade	Freshman	58	21.0
	Sophomore	66	23.9
	Junior	72	26.1
	Senior	54	19.6
	Master’s and above	26	9.4
Major category	Science/Engineering/Agriculture/Medicine	98	35.5
	Economics/Management/Law	85	30.8
	Humanities and Social Sciences	42	15.2
	Arts and Sports	31	11.2
	Other	20	7.3
Place of origin	Urban	102	37.0
	County towns and townships	94	34.0
	Rural	80	29.0
Annual family income	Below RMB 50,000	62	22.5
	RMB 50,000–100,000 (excluding RMB 100,000)	108	39.1
	RMB 100,000–200,000 (excluding RMB 200,000)	74	26.8
	Above RMB 200,000	32	11.6
Taken entrepreneurship course	Yes	162	58.7
	No	124	41.3
Entrepreneurial experience	Yes	89	32.2
	No	187	67.8

### 3.3 Data analysis

To ensure credibility of the empirical analysis and the validity of the research inferences, this study used SPSS 31.0 and AMOS 28.0 to implement multidimensional data preprocessing and quality inspection, including common method bias tests, reliability analysis, and validity analysis, in order to remove the interference of non-systematic error, and subsequently to provide true and reliable data support for the estimation of various parameters in the structural equation model. Common method bias refers to artificially inflated correlations among variables due to the same method used to measure all variables, a frequently observed phenomenon in self-report questionnaire research (48). Since all four variables in this study (digital technology application, social entrepreneurial intention, psychological capital, and digital health practice literacy) used self-evaluation as a measurement, potential common method bias needed to be tested. Following the statistical logic of Harman’s single-factor test, this study systematically examined common method bias (CMB) in the sample data. If the cumulative variance explained by a single principal component is substantially higher than that of the other factors—typically with 40% used as the threshold—serious common method bias may exist in the data (49). The results showed that three factors had eigenvalues greater than 1, and the variance explained by the first factor was 12.826%. According to the criterion for identifying common method bias, the data therefore do not exhibit a serious common method bias problem. No single dominant factor was found, indicating that the sample data have good independence and are suitable for subsequent analysis.

Since the core variables in this present study were measured by established instruments, assessing the reliability of the instruments was an important first step in ensuring the power and applied significance of the later experimental work. We began reliability testing, in particular, with Cronbach’s alpha which measures the homogeneity and internal consistency of the items in each scale. In general, this statistic will fall between 0 and 1. Cronbach’s alpha values above 0.8 suggest good internal consistency, values between 0.7 and 0.8 are satisfactory, and values lower than 0.7 indicate that some items on the scale need to be revised or deleted. As shown in Table 2, the Cronbach’s alpha coefficients for social entrepreneurial intention, digital technology application, psychological capital and digital health practice literacy were 0.916, 0.938, 0.962 and 0.981, respectively, all of which significantly exceeded the threshold of 0.8 and reached the minimum levels for high reliability, indicating high internal consistency and structural stability of the measurement instruments and providing a valid and reliable quantitative basis for analyzing the relationship between digital technology application and university students’ social entrepreneurial intention.

**Table 2:** Reliability analysis.

Variable	Cronbach’s *α*	Number of Items
Social entrepreneurial intention	0.916	5
Digital technology application	0.938	4
Psychological capital	0.962	25
Digital health practice literacy	0.981	21

To further examine the structural validity and check the extent to which items map onto their underlying constructs, EFA was conducted in the present study on the items, prior to confirmatory task presented earlier. The data were examined using exploratory factor analysis (EFA), with Principal Component Analysis to extract factors, followed by varimax rotation to improve the factor-loading matrix to maximize distinctiveness of the dimensions for assignment of factors. Suitability for factor analysis is typically judged by the Kaiser–Meyer–Olkin (KMO) measure and Bartlett’s test of sphericity. The KMO statistic measures partial correlations among variables and ranges from 0 to 1; the closer the value is to 1, the more prominent the common characteristics among factors. Bartlett’s test is used to determine whether the correlation matrix among variables is an identity matrix; a significant result (*p* < 0.001) indicates that the variables are sufficiently correlated for factor analysis (50). As shown in Table 3, the KMO value was 0.983, well above the acceptable threshold of 0.8, and the significance value was 0.000, which is below 0.001. These results indicate excellent clustering and correlation within the sample data and show that the internal structure of the variables fully meets the requirements for factor analysis.

**Table 3:** KMO and Bartlett’s test.

Indicator	Value
Kaiser–Meyer–Olkin measure of sampling adequacy	0.983
Approximate chi-square of Bartlett’s test of sphericity	16555.905
df	1,485
Sig.	0.000

**Figure 2:**
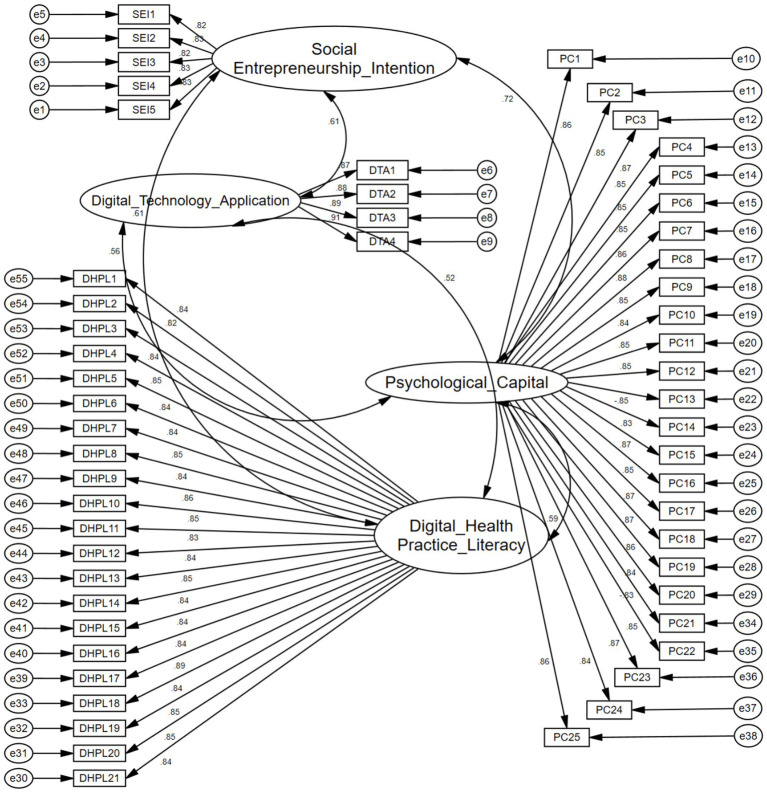
Confirmatory factor analysis (CFA) model.

After the preliminary EFA examination, this study further employed confirmatory factor analysis (CFA) to rigorously verify the degree of fit between the prespecified measurement model and the observed sample data through structural equation modeling. In AMOS 28.0, a first-order multidimensional correlational model was created, which included the four core variables, as illustrated in Figure 2, providing the structural basis needed for accurately conducting the path analysis that follows.

The specific results are exhibited in Table 4. Firstly, at the item-observation level, all the items tapping the latent variables had very robust loadings on their relevant factors. The standardized factor loadings of the items for social entrepreneurial intention, digital technology application, psychological capital, and digital health practice literacy were in the ranges of 0.820–0.830, 0.870–0.910, 0.830–0.880, and 0.820–0.900, respectively. These values not only clearly exceed the statistical threshold of 0.5 but are also highly significant at *p* < 0.001, fully confirming the strong representational capacity of the observed indicators for their corresponding theoretical constructs. Second, in terms of convergent validity, the composite reliability (CR) and average variance extracted (AVE) values of all core variables performed very well.


$$l_{\mathrm{AVE}}=\frac{\left(\sum \lambda^{2}\right)}{Ν}$$

$$l_{\mathrm{CR}}=\frac{{\left(\sum \lambda \right)}^{2}}{{\left(\sum \lambda \right)}^{2}+\sum \varepsilon }$$

**Table 4:** Results of the confirmatory factor analysis.

Item	Variable	Standardized factor loading	AVE	CR	Unstandardized factor loading	S.E.	C.R.	*p*
SEI1	Social entrepreneurial intention	0.822	**0.687**	**0.916**	1			
SEI2		0.832			1.009	0.061	16.543	***
SEI3		0.823			0.967	0.059	16.278	***
SEI4		0.835			1.096	0.066	16.617	***
SEI5		0.831			0.997	0.061	16.228	***
DTA1	Digital technology application	0.874	**0.792**	**0.938**	1			
DTA2		0.884			1.017	0.049	20.569	***
DTA3		0.894			1.001	0.048	21.007	***
DTA4		0.908			1.094	0.05	21.679	***
PC1	Psychological capital	0.864	**0.730**	**0.980**	1			
PC2		0.854			0.983	0.05	19.547	***
PC3		0.868			1.007	0.05	20.177	***
PC4		0.854			0.976	0.05	19.535	***
PC5		0.848			0.928	0.048	19.257	***
PC6		0.854			0.977	0.05	19.549	***
PC7		0.855			1.026	0.052	19.581	***
PC8		0.875			1	0.049	20.515	***
PC9		0.854			1.02	0.052	19.549	***
PC10		0.836			0.956	0.051	18.756	***
PC11		0.846			0.893	0.046	19.199	***
PC12		0.853			0.962	0.049	19.479	***
PC13		−0.852			−0.976	0.05	−19.427	***
PC14		0.830			0.946	0.051	18.505	***
PC15		0.872			1.008	0.05	20.343	***
PC16		0.85			0.997	0.052	19.345	***
PC17		0.872			1.003	0.049	20.362	***
PC18		0.874			1.042	0.051	20.427	***
PC19		0.856			1.008	0.051	19.628	***
PC20		0.838			0.937	0.05	18.834	***
PC21		−0.830			−0.972	0.052	−18.515	***
PC22		0.854			0.978	0.05	19.529	***
PC23		0.872			0.982	0.048	20.341	***
PC24		0.840			0.963	0.051	18.914	***
PC25		0.861			0.936	0.047	19.838	***
DHPL1	Digital health practice literacy	0.836	**0.714**	**0.981**	1			
DHPL2		0.824			0.966	0.055	17.492	***
DHPL3		0.842			0.985	0.054	18.148	***
DHPL4		0.846			0.931	0.051	18.317	***
DHPL5		0.843			0.961	0.053	18.185	***
DHPL6		0.844			0.938	0.051	18.252	***
DHPL7		0.850			0.986	0.053	18.477	***
DHPL8		0.837			0.945	0.053	17.974	***
DHPL9		0.856			1.004	0.054	18.687	***
DHPL10		0.853			1.007	0.054	18.562	***
DHPL11		0.832			0.933	0.052	17.798	***
DHPL12		0.841			0.96	0.053	18.118	***
DHPL13		0.850			0.944	0.051	18.456	***
DHPL14		0.840			0.957	0.053	18.098	***
DHPL15		0.838			0.906	0.05	18.023	***
DHPL16		0.837			0.982	0.055	17.988	***
DHPL17		0.885			1.04	0.052	19.872	***
DHPL18		0.841			0.998	0.055	18.137	***
DHPL19		0.853			1	0.054	18.569	***
DHPL20		0.850			0.993	0.054	18.46	***
DHPL21		0.843			0.927	0.052	17.955	***

***indicates *p* < 0.001. Bold entries signify primary construct-level fit indices, differentiated from item-level estimates.

Specifically, social entrepreneurial intention (AVE = 0.687, CR = 0.916), digital technology application (AVE = 0.792, CR = 0.938), psychological capital (AVE = 0.730, CR = 0.980), and digital health practice literacy (AVE = 0.714, CR = 0.981) all exceeded the widely accepted benchmark values in the literature (AVE > 0.5, CR > 0.7). These statistics collectively indicate that the measurement scales used in this study possess excellent convergent consistency and component validity, thereby providing a high-confidence measurement basis for the inference of paths among variables.

Discriminant validity was assessed to confirm that each latent construct is statistically distinct from the others, meaning that the correlation between any construct and the remaining constructs should be lower than the internal cohesiveness of its own indicators. This study employed the classic Fornell–Larcker criterion to test discriminant validity. According to this criterion, the absolute value of the standardized correlation coefficient between any two constructs must be smaller than the arithmetic square root of the corresponding construct’s AVE value (51). Pearson correlation analysis was first conducted to estimate the standardized correlation coefficients among the four core variables, as shown in Table 5. The findings also show a high degree of theoretical consistency within the system of path relationships. In particular, the correlation coefficient between digital technology application and social entrepreneurial intention was found to be 0.608, suggesting that deeper embedding of technology leads to a more positive catalysing effect on students’ motivation towards entrepreneurship. Digital technology application was also significantly and positively related to psychological capital (*r* = 0.557) and digital health practice literacy (*r* = 0.520), indirectly supporting the empowering role of digital technology application as a “scaffold” for individuals’ psychological stability and digital capability. Furthermore, the correlations between social entrepreneurial intention, psychological capital, and digital health practice literacy were 0.724, 0.611 and 0.591 respectively, and these close covarying relationships provide exploratory grounds for the subsequent analysis of multiple mediating effects. Next, we calculate the square root of the AVE value: social entrepreneurial intention = 0.829, digital technology application = 0.890, psychological capital = 0.855, digital health practice literacy = 0.845, and then compared with each other and obtained that the two-factor standardized correlation coefficients of all factors are less than the square root of the AVE value of the corresponding factors. This pattern provides strong evidence that the latent variables in our model show excellent exclusivity and discriminant validity, excluding a serious construct-confounding bias, and showing that each scale captures its intended research dimension independently and precisely.

**Table 5:** Discriminant validity test across dimensions.

Variable	Social entrepreneurial intention	Digital technology application	Psychological capital	Digital health practice literacy
Social entrepreneurial intention	**0.687**			
Digital technology application	0.608	**0.792**		
Psychological capital	0.724	0.557	**0.730**	
Digital health practice literacy	0.611	0.520	0.591	**0.714**
Square root of AVE	0.829	0.890	0.855	0.845

Bold diagonal values report the square root of the average variance extracted (AVE) per construct.

### 3.4 Results analysis

**Figure 3:**
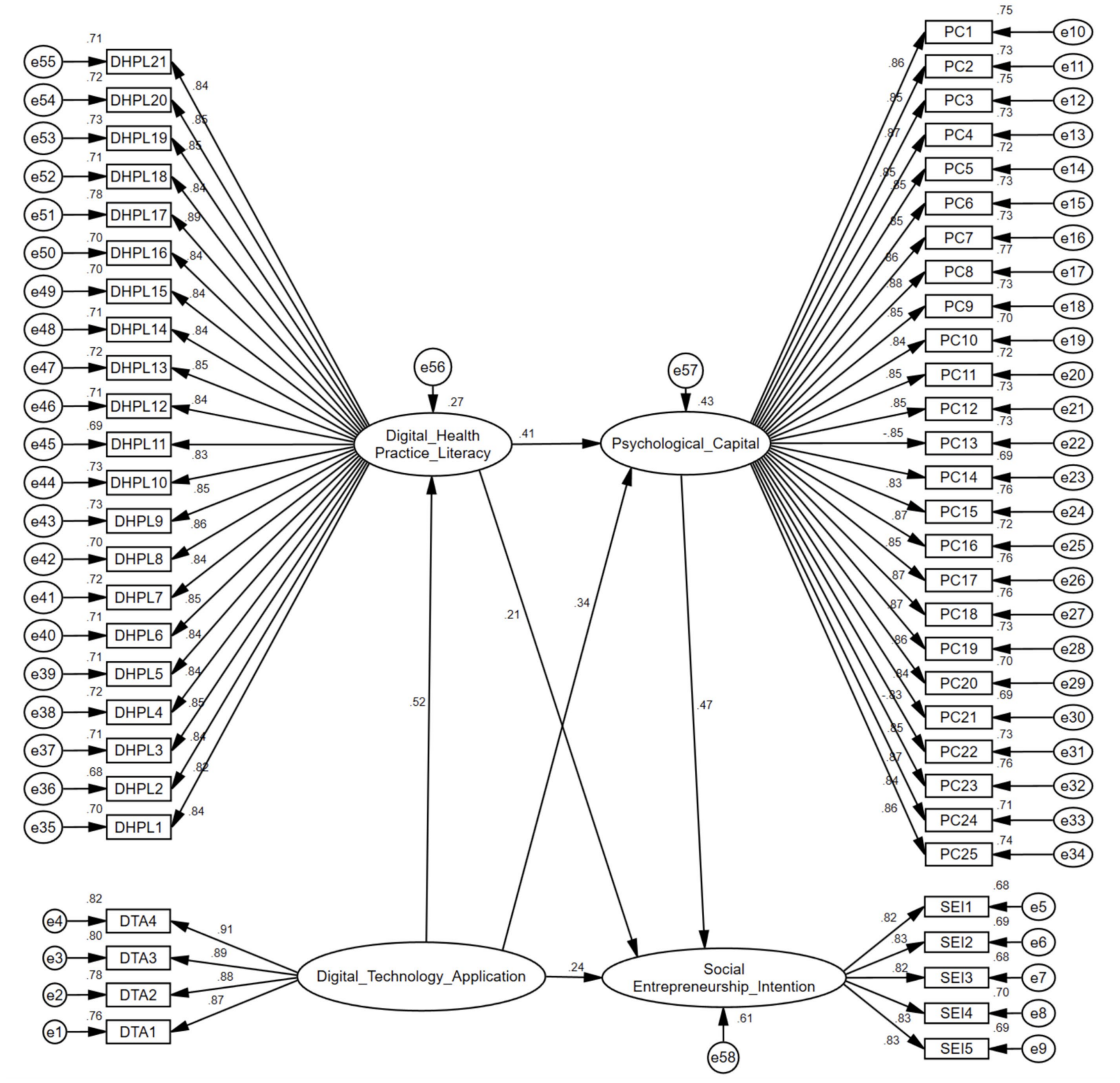
Standardized structural equation model.

Using the software AMOS 28.0, we build a structural equation model and test the relationships among digital technology application, digital health practice literacy, psychological capital, and social entrepreneurial intention. Based on the conceptual model from the hypotheses above, we fit and revise our questionnaire data, and so the final path relationships are summarised in the following revised standardised model. Figure 3.

The goodness-of-fit of the model above, the SEM implementation of the hypotheses must be considered, for practical purposes, as the essential step in the evaluation of whether the theoretical model exists as fit with the covariance matrix of the sample. The eminence of the statistical basis of the path coefficients internal to the SEM and the entirety of the scientific face validity end of the multiple mediation rests on this. Following the fit-index framework applied in studies on organizational behavior and psychology, we empirically examined the associations between digital technology application, digital health practice literacy, psychological capital and university students’ social entrepreneurial intention. We used a combined set of indices - the chi-square/degrees-of-freedom, absolute fit and a number of incremental fit indices - to assess the overall model fit. The results are reported in Table 6. As shown in the table, all key indicators fell within acceptable ranges. The chi-square/degrees-of-freedom ratio (CMIN/DF) was 1.021, and values between 1 and 3 generally indicate excellent model fit; a value of 1.021 is extremely close to 1, indicating that the model structure is parsimonious and highly explanatory with respect to the data. The RMSEA was 0.009, substantially below the critical threshold of 0.05. The incremental fit indices RFI, IFI, CFI, NFI, and TLI were 0.915, 0.998, 0.998, 0.918, and 0.998, respectively, all above the accepted standard of 0.9. These findings indicate that the multiple mediation model developed in this study fits the observed survey data very well and provides a scientific basis for subsequent path estimation and hypothesis testing.

**Table 6:** Model fit indices.

Indicator	CMIN	DF	CMIN/DF	RMSEA	RFI	IFI	CFI	NFI	TLI
Acceptable range			< 3	< 0.05	> 0.9	> 0.9	> 0.9	> 0.9	> 0.9
observed value	1453.296	1,424	1.021	0.009	0.915	0.998	0.998	0.918	0.998

On the premise of good model fit, this study used the path coefficients in the structural equation model to examine the direct effect of digital technology application on university students’ social entrepreneurial intention, as well as the mediating path effects of digital health practice literacy and psychological capital between the two. Path coefficients directly reflect the strength of the influence relationships among variables, while the critical ratio (C.R.) is used to test the significance of path coefficients. The specific results are shown in Table 7. This study first examined the direct effect of digital technology application on college students’ social entrepreneurial intention. The unstandardized path coefficient from digital technology application to social entrepreneurial intention was 0.187, with a significance level of *p* < 0.001. The path coefficient was significantly positive, and Hypothesis H1 was verified: the higher the degree to which college students apply digital technology, the stronger their social entrepreneurial intention. The test results for the path “digital technology application → digital health practice literacy → social entrepreneurial intention” showed that digital technology had a significant positive predictive effect on digital health practice literacy, with an unstandardized path coefficient of 0.424 and p < 0.001, while the effect of digital health practice literacy on social entrepreneurial intention also reached a significant level, with an unstandardized path coefficient of 0.203. Thus, Hypothesis H2 was verified, and it also revealed the logical closed loop of “promoting learning through use”: in the process of frequently applying digital tools, college students not only master specific software operations, but also subtly improve core digital health practice competencies such as data sensitivity, digital collaboration ability, and crisis management. According to the research hypothesis, H3 includes two sub-paths: the unstandardized path coefficient from digital technology application to psychological capital was 0.311, with a C. R. value of 5.787 (greater than 3.29) and *p* < 0.001, and the path coefficient was significantly positive, indicating that the application of digital technology had a significant positive effect on individual psychological capital; the unstandardized path coefficient from psychological capital to social entrepreneurial intention was 0.401, indicating that by enhancing psychological capital, college students can provide a necessary emotional buffer for the relatively high-risk behavior of entrepreneurship, making them more inclined to make positive entrepreneurial decisions, thus supporting Hypothesis H3. To further explore the stepwise influence pattern among variables, Hypothesis H4 in this study proposed a chain mediating effect along the path “digital technology application → digital health practice literacy → psychological capital → social entrepreneurial intention.” As shown in Table 7, the path coefficient for “digital technology application → digital health practice literacy” had already been verified as 0.424 (p < 0.001), and the path coefficient for “psychological capital → social entrepreneurial intention” had already been verified as 0.401 (p < 0.001). The path coefficient for the key sub-path “digital health practice literacy → psychological capital” was 0.461, with a significance level of p < 0.001, and the path coefficient was positive, indicating that digital health practice literacy had a certain positive effect on psychological capital. Therefore, Hypothesis H4 was preliminarily supported.

**Table 7:** Structural equation model path coefficients.

Path	Unstandardized coefficient	S.E.	C.R.	*p*	Standardized coefficient
Digital health practice literacy—Digital technology application	0.424	0.049	8.611	***	0.520
Psychological capital—Digital technology application	0.311	0.054	5.787	***	0.342
Psychological capital—Digital health practice literacy	0.461	0.067	6.926	***	0.413
Social entrepreneurial intention—Digital health practice Literacy	0.203	0.055	3.727	***	0.212
Social entrepreneurial intention—Digital technology application	0.187	0.044	4.193	***	0.239
Social entrepreneurial intention—Psychological capital	0.401	0.054	7.494	***	0.466

***indicates *p* < 0.001.

To further test the predominant influence structure of the variables, we performed a Bootstrapping repeated sampling procedure believed to be promoted by Preacher and Hayes (52) for robustness test of the same mediating effects. Because the respective confidence intervals are believed to be narrower and more reliable - some would say more powerful - the literature have gravitated towards this method. Please see path coefficients and respective test values herein in Table 8. The results show that the mediating effect of the path “digital technology application → digital health practice literacy → social entrepreneurial intention” was 0.086, with a Bootstrap standard error (BootSE) of 0.026 and a 95% confidence interval of [0.040, 0.142], which does not include 0. This indicates that the independent mediating effect of digital health practice literacy is significant and provides further support for H2. The mediating effect of the path “digital technology application → psychological capital → social entrepreneurial intention” was 0.125, with BootSE = 0.027 and a 95% confidence interval of [0.082, 0.190], which likewise does not include 0, confirming the significant independent mediating role of psychological capital and further supporting H3. The standardized effect of the serial path “digital technology application → digital health practice literacy → psychological capital → social entrepreneurial intention” was 0.078, with a 95% confidence interval of [0.051, 0.117], again excluding 0. This result further confirms H4.

**Table 8:** Results of the mediation effect test.

Path	Mediating effect	BootSE	BootLLCI	BootULCI
Digital technology application—Digital health practice literacy—Social entrepreneurial intention	0.086	0.026	0.040	0.142
Digital technology application—Psychological capital—Social entrepreneurial intention	0.125	0.027	0.082	0.190
Digital technology application—Digital health practice literacy—Psychological capital—Social entrepreneurial intention	0.078	0.017	0.051	0.117

## 4 Conclusions and discussion

### 4.1 Research conclusions

Based on social innovation theory, this study systematically reveals the underlying mechanism through which the application of digital technology drives social entrepreneurial intentions in the field of health among university students, through structural equation modeling (SEM) analysis of 276 valid samples and Bootstrap path testing, and draws the following core conclusions:

First, digital technology application is a core driving force stimulating university students’ social entrepreneurial intention in the healthcare field. The results show that digital technology application has a significant and positive direct effect on social entrepreneurial intention, with an unstandardized path coefficient of 0.187 and *p* < 0.001, thereby supporting H1. From the perspective of social innovation theory, digital technology is positioned not just as a productivity tool in general entrepreneurship, but as a critical enabler that can trigger social innovation in health systems (53). With macro conditions leading to a slowdown of the economy, traditional social entrepreneurship in healthcare often requires very high thresholds of resource acquisition and faces severe information asymmetry between medical providers and underserved populations. The accessibility of digital technology penetrates these barriers and allows for the decentralized recombination of resources for health-focused social innovation. For university students on the cusp of entrepreneurship, digital technology becomes an all-purpose innovation tool for addressing public health challenges: generative AI helps students create high-quality health intervention business plans, big-data analytics tools help students accurately identify social pain points in medical resource distribution and health service accessibility that are often neglected by mainstream markets (54), and low-code or no-code tools help students not otherwise technically educated build digital health platforms. All of this technology lowers the marginal cost of launching health ventures for students and helps to shrink the perceived boundary of effort required by students to take the leap of faith to become entrepreneurs trying to solve wicked problems like health inequity in rural areas, barriers to chronic disease management for low-income populations, and the imbalance between burgeoning digital health demands and constrained public health infrastructures. As Bokhari and Myeong (55) propose that AI will change the logic of individual decision-making, and that this technology will facilitate the intention of university students to convert social ideality of health equity into practice, then the greater the intensity of AI use by university students, the more sensitive they are to social observation and action social health disparities and gaps in primary healthcare coverage, and the greater their intention to launch ventures that improve population health outcomes.

Second, digital health practice literacy played a key capability-mediated role between digital technology application and social entrepreneurial intention in the health sector. The path analysis results showed that digital technology application significantly and positively predicted digital health practice literacy (*β* = 0.424, p < <0.001), while digital health practice literacy also effectively drove social entrepreneurial intention (β = 0.203, p < <0.001), providing empirical support for research hypothesis H2. This conclusion reveals the pathway through which the logic of empowerment is realized in social innovation for healthcare. Social innovation theory emphasizes that an individual’s capacity for change stems from deep mastery of the tools of production relevant to their targeted social domain (20). The statistical data indicate that AI cannot completely and directly replace human clinical or public health judgment; rather, through a mechanism of “learning through use,” it compels university students to accumulate their foundational digital capabilities in the process of interacting with health-specific digital technologies such as AI-driven symptom checkers, telemedicine platforms, and electronic health record systems. This stepwise growth in digital health practice literacy provides the capability assurance that enables individuals to extract “social entrepreneurial opportunities” from complex health phenomena—such as identifying underserved patient populations, designing community-based preventive care models, and leveraging wearable health data for population-level interventions. This study argues that digital health practice literacy plays an important mediating role in the model, transforming external technological dividends into logical analytical ability and digital governance capacity that individuals can mobilize at any time to address public health challenges. Therefore, the strength of university students’ intention to engage in social entrepreneurship in the health sector depends to a large extent on whether they can, through the frequent use of digital technologies such as AI, internalize technological advantages into stable digital survival skills that are directly applicable to navigating healthcare ecosystems, regulatory environments, and patient-centered care models.

Third, psychological capital plays an important mediating role in the process by which digital technology application drives social entrepreneurial intention toward health innovation. Path analysis showed that digital technology application positively affects psychological capital (*β* = 0.311, *p* < 0.001), and psychological capital exerts a significant positive catalytic effect on entrepreneurial intention (β = 0.401, p < 0.001), thereby supporting H3. This finding demonstrates that digital technology application has the function of a “psychological scaffold” in the arena of health-focused social entrepreneurship. When university students face complex health inequities—such as disparities in maternal health outcomes, uneven distribution of specialist care across regions, or the stigma surrounding mental health services in underserved communities—the simulation, forecasting, and instant-feedback functions provided by digital health technologies quietly offer them a layer of “digital buffering” against the emotional toll of confronting entrenched public health crises (68). This sense of security enhances their psychological capital so that when facing the high-risk decision of launching a healthcare venture—whether navigating strict medical regulatory frameworks, securing patient trust, or managing the long R&D cycles typical of health interventions—they present stronger resistance to stress and more optimistic expectations. As Carter et al. (56) point out, “in the AI era, this sense of technological support is transforming into individuals’ positive psychological energy,” and the logic through which digital technology application drives health entrepreneurship is thus not just a logic of single technological deduction, but also a logic of expanding psychological energy through technological empowerment in the face of formidable health system challenges, and the stability of psychological capital directly guards the persistence of social entrepreneurial intention when students must sustain motivation through the prolonged and uncertain journey of achieving meaningful health impact.

Fourth, the serial mediation path from digital health practice literacy to psychological capital underpins the formation of college students’ entrepreneurial intention within the digital public health field. The core finding of this study reveals a serial mediating mechanism: digital technology application improves digital health practice literacy, further boosting psychological capital and consequently promoting social entrepreneurial intention. The value of the mediating effect of this serial path was 0.078, and its 95% confidence interval does not include 0, supporting H4. This shows that under the influence of digital technology application, the mechanism of college students’ social entrepreneurial intention in healthcare has changed from single-path channel into diversified co-acting mechanism, forming a technology–capability–mindset–action close-loop transmission process specifically oriented toward public health innovation. The logic lies in that the deep application of digital health technology as an external triggering indicates to redefine the boundary of individual capability through the guidance of steps upgrading of digital health practice literacy. After mastering intelligent tools for health data analysis, remote patient monitoring, and AI-assisted diagnosis, university students shift their assessment of social entrepreneurship in healthcare from “uncontrollable” to “controllable and predictable” —viewing health system gaps not as insurmountable institutional failures but as addressable market opportunities for social impact and this increase of capability directly feeds back into psychological capital (*β* = 0.461, *p* < 0.001). This is the crosswise end of the major leap between an expansion of capability and an enhancement of mindset in the health sector. In this serial mediation, we find that in the digitally intelligent health environment, university students are no longer tool users; rather, through mutual reinforcement of digital health practice literacy and psychological capital, they reconstitute themselves as social innovators capable of redesigning care delivery models, democratizing health information, and pioneering community-based digital health interventions (57). This coordinated evolution of mentality and capability is a high-gain positive reinforcement feedback loop for the very reason that it prepares the individual well to maintain a state of productive tension with complex challenges of health social entrepreneurship —from scaling telehealth in rural communities to ensuring equitable AI health algorithms that do not exacerbate existing disparities.

### 4.2 Theoretical contributions

This study, grounded in the framework of social innovation theory, deconstructs and reconstructs the driving mechanisms behind college students’ social entrepreneurial intentions. Its theoretical contribution lies not only in confirming the direct catalytic role of digital technology application, but also in introducing the dual dimensions of digital health practice literacy and psychological capital, thereby accurately depicting the dynamic evolutionary process through which individuals transition from technological exposure to addressing public health challenges. These findings supplement existing research on the interaction between digital health intervention technologies and individual micro-level psychological resources, and provide a valuable theoretical reference for understanding how college students act as digital health innovators in the era of digital public health.

First, this study expands and deepens the micro-level explanatory power of social innovation theory in the field of public health, achieving a paradigm shift from macro-level policy intervention to micro-level individual entrepreneurial evolution. Traditional research on public health innovation has long focused on macro institutional changes and social intervention policy design (2, 20, 22). Under a structural determinism perspective, individuals’ micro-level innovative agency is often overshadowed by macro frameworks (58). This study shifts the analytical focus to college students as micro-level actors and empirically verifies the driving effect of digital technology application on individual social entrepreneurial intention. It demonstrates that, in the digital health era, the momentum of social innovation has shifted toward digitally skilled youth, providing empirical support for the psychological and behavioral foundations of health innovation theory. Furthermore, based on the chain mediation of “digital health practice literacy → psychological capital,” this study clarifies that social health innovation involves both external transformation of public health issues and internal reconstruction of psychological resilience under digital empowerment (59), completing the theoretical linkage from macro intervention to micro psychology and establishing college students as autonomous observers and initiators of health action.

Second, the study refines the internal logic of digital technology empowerment in health entrepreneurship and addresses the gap in research on the interaction between digital health intervention and psychological capital. Existing studies often treat digital technology as homogeneous medical resource input, emphasizing its direct relationship with health outcomes while overlooking the key process of individual cognitive transformation (60), and disconnecting digital tools from micro-level resilience. By introducing digital health practice literacy and psychological capital as dual mediators, this study empirically confirms both parallel and chain mediation effects, clearly illustrating the transmission path of “digital technology application—digital health practice literacy—psychological capital—social entrepreneurial intention.” It identifies digital health practice literacy as the capability foundation and psychological capital as the cognitive core, revealing how improvements in digital literacy enhance psychological resilience and addressing the question of how digital health tools empower deep psychological and behavioral change (61). This forms a closed theoretical loop of “external digital environment—internal capability and psychological reconstruction—health entrepreneurship generation.”

Finally, the study enriches the strategic connotation of digital health practice literacy in the public health context and extends its theoretical boundaries in health-related social innovation. Traditional research tends to confine digital health literacy to basic cognitive abilities of accessing and understanding health information, without fully exploring its strategic role in intervention practice and solution innovation. This study elevates digital health practice literacy into a higher-level practical model that enables individuals to identify and address public health issues using digital tools (60). Individuals with high digital health practice literacy can transform complex health inequalities into actionable digital governance targets, serving as a bridge between health intervention vision and implementation (62). The study further defines digital health practice literacy as a core capital for value creation in social innovation (58), showing its role in enabling college students to participate in public health governance and aligning digital capability with a sense of public health mission.

### 4.3 Practical implications

Based on the empirical findings of this study, and considering the practical intervention needs in the current digital public health sector, this study provides important practical implications from three dimensions: health innovation education in higher education institutions, government public health policy support, and reshaping individual literacy of college students.

First, universities should move beyond the traditional single-track model of generic computer training and build a concrete, three-tiered interdisciplinary curriculum system that integrates “digital health applications + social innovation + hands-on intervention.” Empirical research shows that the chain pathway between digital health practical literacy and psychological capital is the underlying driving force behind the formation of individual social entrepreneurial intention (H4). Moreover, most university students’ capacity to respond to complex health challenges and their resilience stem from schools’ targeted educational interventions and literacy cultivation (13). Therefore, innovation education in universities must not remain at the superficial level of merely teaching students how to operate digital devices. Future educational designs should incorporate at least the following three actionable components: (i) Specialized “digital health innovation” courses. Universities should offer dedicated courses that move beyond basic software instruction to focus on applied digital health competencies, for instance, AI-assisted symptom analysis, telemedicine platform architecture, electronic health record analytics, and wearable health data interpretation. Such coursework directly enhances students’ digital health practice literacy (aligning with H2) by providing structured, hands-on engagement with the tools that drive modern public health interventions, rather than treating digital literacy as an isolated technical add-on. (ii) Structured social entrepreneurship training programs targeting health equity challenges. Higher education institutions should establish incubators or certificate programs that guide students through the full cycle of health social venture development: identifying underserved patient populations, designing community-based preventive care models, navigating medical regulatory frameworks, and securing ethical approvals for digital health pilots. Crucially, these programs should explicitly embed psychological capital-building modules—such as resilience training, stress-inoculation workshops, and mentorship networks tailored to the high-risk, long-R&D-cycle nature of healthcare ventures—thereby addressing the psychological barriers that deter students from launching social health enterprises (aligning with H3). (iii) AI-assisted medical service design workshops. Universities should collaborate with local public health agencies and clinical partners to host interdisciplinary, problem-based workshops where mixed teams of students, clinicians, and technologists co-design solutions for concrete health disparities (e.g., maternal health gaps in rural regions, mental health access barriers for low-income communities, or chronic disease monitoring in aging populations). These workshops function as “low-cost public health testing grounds” and “digital shock absorbers,” allowing students to simulate intervention strategies under guided, low-risk conditions. By iteratively prototyping AI-driven diagnostic aids or mobile health applications, students internalize digital health tools into actionable governance skills while simultaneously strengthening their psychological capital through manageable experimentation (aligning with H1 and H4). Through this three-tiered integration, students come to recognize that digital technology is not merely an office plug-in, but a cognitive lever capable of advancing health equity. When literacy improves through concrete coursework, and psychological capital is fortified through simulated practice, entrepreneurship education achieves a systematic elevation from passive knowledge transmission to active, stress-resilient mindset reshaping.

Second, at the government level, efforts should be made to strengthen the inclusiveness and public intervention orientation of digital health technologies and to build a multidimensional support system. Based on the findings of H1 and H2, the level of technological application and digital health practice literacy constitute the entry qualifications for young people to participate in health governance. Therefore, the government and relevant public health authorities should carry out deep optimization on the policy supply side, giving priority to ensuring the inclusive opening of open-source health datasets and medical-assistance APIs to university student social entrepreneurship groups, so that every socially responsible young person can fairly access digital innovation tools. The government can formulate explicit incentive policies guided by “public health value orientation” and introduce special grants or credit-enhancement measures specifically for “digitally driven grassroots health collaboration and intervention projects” (63). For example, it can encourage university students to use digital applications to improve the efficiency of community chronic disease management and to promote the balanced and scalable application of mental health service resources (64). By establishing a “Digital Health Social Innovation Incubation Competition” and providing outstanding public health startups with a fast-track review channel, the government can not only transform students’ digital capabilities but also stimulate their intrinsic sense of social responsibility. The government can also work in coordination with medical and health institutions to establish analysis platforms based on cutting-edge digital intelligence, using big data to provide university students with accurate forecasts of community health needs and policy explanations.

Third, from the individual college student perspective, resilient thinking should be cultivated in the context of health crises, enabling a shift in identity from passive technology users to improvers of public health. College students should deeply recognize that the application of digital technology is by no means merely an information retrieval tool, but rather a powerful weapon for addressing complex social health challenges (19). In daily practice, students should proactively move beyond the trap of using digital devices only for basic entertainment and strive to deeply embed digital technology into social health insight and the simulation of intervention strategies. This frequent, interactive technological learning is not only intended to improve literacy, but also to internalize the ability to process massive and complex health data into a sense of self-efficacy for coping with potential psychological crises and external pressures (65). When individuals acquire the capacity to use digital tools to structure interventions addressing social health inequalities, their internal psychological capital is fundamentally strengthened. In response to the loss of intention caused by the high-risk nature of the health intervention field, college students should learn to use digital technology to conduct full-scenario public health risk simulations, maintain a dispositional optimism and resilience, and transform feelings of difficulty and fear into a sense of competence in social intervention empowered by technology. In the digital era, the highest form of competitiveness is an individual’s ability to use technology to bridge social health gaps. College students should actively consider how to use the algorithmic tools at their disposal to address pain points in the care of special populations or in grassroots lifestyle interventions (66); this kind of intention grounded in a sense of public health mission and personal resilience is the most vital force in social entrepreneurship.

### 4.4 Research limitations and future prospects

Although this study constructs a rigorous chain model of “digital technology application—digital health practice literacy—psychological capital—social entrepreneurial intention,” it is constrained by objective conditions and still has the following limitations, which point the way for future research. First, geographic concentration and sample size may limit generalizability. The survey primarily focuses on the Yangtze River Delta region (N = 276), where digital healthcare resources are relatively abundant. This may overlook the “digital health divide” faced by university students in less developed regions and constrains the generalizability of the findings to broader populations. Future research should expand the sampling scope and systematically examine the heterogeneous effects of grassroots public health infrastructure on digital technology empowerment through cross-regional empirical comparisons. Second, cross-sectional design and self-reported data restrict causal inference and may introduce bias. Cross-sectional data struggle to capture the dynamic, long-term impact of digital health tools on individual psychological resources. Additionally, the exclusive reliance on self-reported questionnaires may introduce common method variance and social desirability bias. Future research could adopt longitudinal tracking designs with multi-time-point observations to deeply explore the intervention trajectory of digital technology on psychological resilience and entrepreneurial decision-making. Incorporating multi-source data—such as behavioral records, peer evaluations, or objective digital engagement metrics—would also help triangulate self-report measures and strengthen causal claims. Finally, the model lacks exploration of the macro public health context. External environmental variables driving health social innovation are far more diverse than those currently included. Future research could introduce moderating variables such as “perceived health vulnerability” or “community-level healthcare policy support” to explore the boundary conditions under which digital technology drives university students’ health social entrepreneurial intentions amid varying epidemiological risks and community resource constraints, thereby constructing a more comprehensive public health social innovation model.

## Data Availability

The raw data supporting the conclusions of this article will be made available by the authors, without undue reservation.
